# Technology Enhanced Health and Social Care for Vulnerable People During the COVID-19 Outbreak

**DOI:** 10.3389/fnhum.2021.721065

**Published:** 2021-09-10

**Authors:** Evangelia D. Romanopoulou, Vasiliki I. Zilidou, Sotiria Gilou, Ioanna Dratsiou, Annita Varella, Vasileia Petronikolou, Aikaterini-Marina Katsouli, Maria Karagianni, Panagiotis D. Bamidis

**Affiliations:** Laboratory of Medical Physics, School of Medicine, Aristotle University of Thessaloniki, Thessaloniki, Greece

**Keywords:** COVID-19, healthcare, ICT, web-based, cognitive training, technology, quality of life, vulnerable people

## Abstract

The COVID-19 pandemic has spread rapidly worldwide with critical consequences in health, as well as in social, economic, and particularly in psychological conditions of vulnerable people, especially older adults. Therefore, it is necessary the direct attention to their health care needs and related interventions. Information and Communication Technology (ICT) have direct impact on older adults’ health and quality of life leading to decreased depression and loneliness, along with empowerment of independent life. Many studies involve cognitive training programs/software based on new technological systems that provide to vulnerable people access to gamified, attractive, cognitive exercises for overall functionality everywhere and at any time. Twenty-four participants (mean age 69.3 years) were assigned to this study. The cognitive training component of LLM Care was used as an interactive software to enhance participants’ cognitive functions. The intervention lasted 12 weeks with the frequency of 2–4 times per week in sessions of at least 30 min. Participants used their personal devices (tablets/laptops) in their own residence, while technical and consulting guidance was provided by LLM Care certified trainers. They were informed about the purpose of the study, while consent forms along with psychological assessments were distributed every 2 weeks to periodically evaluate their psychosocial and mental health conditions. The assessments included the World Health Organization-Five Well-Being Index (WHO-5), the Short Anxiety Screening Test (SAST), the System Usability Scale (SUS) and the Impact Factor Event Scale (IES-R). According to the results, the participants with improved well-being tended to report decreased subjective distress caused by COVID-19, and their engagement with new technologies can potentially minimize the negative outcomes occurred by the current stressful situation, mitigating the effect of hyperarousal symptoms, while increasing their overall well-being. Well-being seems to remain relatively stable among older adults and decreases only when adversities occur, while the usability of the software was perceived as marginally acceptable by participants. The exploitation of the LLM Care contributes to the improvement of older adults’ well-being and alleviates the negative experience caused by stressful situations like COVID-19.

## Introduction

Since the first case of novel coronavirus disease was detected in December 2019, in Wuhan, China (Radwan et al., 2021), COVID-19 outbreak has undoubtedly introduced a new global crisis in economic and socio-political levels with severe consequences in health, as well as in social, economic and psychological condition of people (Radwan and Radwan, 2020). At local level, the first confirmed COVID-19 case in Greece was reported on February 26th, 2020, in Thessaloniki, the second largest city in Greece and right after the Greek Government acted directly imposing containment measures in order to halt the spread of the infection. In particular, proactive controls and restriction measures were taken regionally, including suspension of retail, schools and educational institutes, cultural events and public facilities, while work from home was introduced (Parlapani et al., 2020). During the national lockdown, restriction on public movements throughout the country was enforced, people were allowed to move only for specific purposes and only after they had filled out a special movement document or after having texted a designated number, set out for this purpose by the Greek civil protection (Naftemporiki, 2020; Parlapani et al., 2020).

According to the United Nations Development Program (UNDP), the pandemic should be considered as much more than a health crisis. A pandemic is a public health emergency, a life-threatening condition that disrupts the normal functioning of a community and imposes the sovereignty of particular negative psychological responses like anxiety, fear and uncertainty (Pan American Health Organization (PAHO/WHO), 2009). In this light, it is now well established that the emergence and quick spread of the COVID-19 virus have disrupted practically every facet of daily life, leading to widespread insecurity and scarring overwhelming marks to people (Demetriou et al., 2021). In addition, containment measures resulted in physical and social distancing and self-isolation (World Health Organization (WHO), 2021), which in turn posed a significant impact on the psychological well-being and mental health of people, especially those of older ages (Radwan et al., 2021; World Health Organization (WHO), 2021). Older adults in the age of 60 years or over, due to their weakened immune system, that is often associated with chronic underlying diseases (Meng et al., 2020), present higher infection and mortality rates compared to the general population, while the trends are even more severe for those suffering from other comorbidities and health issues including cardiovascular diseases, chronic respiratory disease, active cancers and diabetes (Kang et al., 2020). In addition, frailty, a state of vulnerability characterized by weakness, progressive declined physiologic function and diminished strength, leading to vulnerability and reduced resilience to stressors with an increased risk of health adverse outcomes (Morley et al., 2013; Kojima, 2016) is highly prevalent in older adults (Kleipool et al., 2018). On the other side, although the prevalence of multimorbidity and frailty increases with age, neither is limited only to older adults (Hanlon et al., 2018). Frailty can also affect younger people (Theou et al., 2012) as it reflects biological and phenotypic, rather than chronological age (Rockwood et al., 2011; Mitnitski and Rockwood, 2016). Since 2001, two general views were created to operationalize frailty clinically: frailty as a clinical syndrome (Fried et al., 2001) or frailty as a state (Mitnitski et al., 2001). In fact, the frailty state is associated with a variety of adverse consequences, such as falls, cognitive decline, infections, hospitalization, disability, institutionalization and death. While pre-frailty classifies a subgroup of individuals at an intermediate stage that predisposes and directly precedes frailty (Fried et al., 2001). Frailty and poor health status at a younger age could partially explain the reason that there are older people who thrive during aging, while some younger adults fail to prosper. Indeed, these findings imply that frailty affect people of any age and may manifest differently in younger and older people (Kehler et al., 2017). According to a recent study, the prevalence of frailty in patients with COVID-19 was similar to that in nursing home residents, while frailty approved an essential prognostic factor for clinicians to predict mortality (Zhang et al., 2021). In this vein, age and frailty, have emerged as critical variables posing a considerable risk for COVID-19 mortality and have resulted in significant emotional disturbances and insecurity, as well as extensive anxiety and depressive disorders to people (Gyasi, 2020; Hewitt et al., 2020).

Aside from the COVID-19’s detrimental effects, especially on vulnerable populations, including older adults and those suffering from chronic diseases like hypertension, cardiovascular disease, diabetes, chronic respiratory disease, and cancer, a lot of discussion was focused on the direct and indirect consequences of the pandemic on people’s psychological health and quality of life. Social isolation and lockdown have put a strain on their economic and social life, as well as their psychological well-being (Serafini et al., 2020). Before the pandemic, the great majority of older individuals were actively engaged in social activities including participation in senior centers and religious activities, traveling and attendance in a variety of other social events. However, all these activities were suspended leading to a certain increase of social isolation and loneliness of older adults (Wu, 2020). Indeed, the disruption of important daily activities and overwhelming feelings of isolation have been shown to have implications on older adults’ health conditions (Santini et al., 2020), decline in cognition, mood and sensitivity to threat (Cacioppo and Hawkley, 2009) alongside a buildup of anxiety and mortality risk (Gorenko et al., 2020). Related studies highlight that 37.1% of older adults had experienced depression and anxiety during the pandemic (Meng et al., 2020), while the emotional response of people aged above 60 years was more apparent as compared to other age groups (Santini et al., 2020). In addition, older adults’ chronic conditions may be intensified as a result of the implications of containment (Qiu et al., 2020) and people with mild cognitive impairment or mild dementia may face a greater challenge during the COVID-19 pandemic (Brooks et al., 2020). Furthermore, dealing with these current and unfamiliar circumstances can be stressful, especially when cognition is compromised (World Health Organization (WHO), 2020), while abstention from participating in face-to-face activities and day care services may impair this population’s cognitive functioning (Braak and Del Tredici, 2012). According to Alzheimer Europe, both people with mild cognitive impairment or mild dementia and their caregivers should be engaged in the following recommendations: building a support network; being well informed; enjoying leisure activities; staying physically and mentally active and keeping socially connected (Alzheimer Europe, 2021).

All things considered, the impact of the current pandemic on psychological well-being and mental health both of older adults (World Health Organization (WHO), 2017) and vulnerable people (United Nations (UN), 2020) is profound and, thus, both sensitization and direct attention to these groups’ health care needs as well as efforts to fulfill their needs adequately are considered essential (Banerjee, 2020). It is imperative that stakeholders and health policymakers join forces to overcome this situation, so that community-dwelling older individuals and people belong in vulnerable groups can receive high-quality, prompt crisis psychological treatments (Yang et al., 2020). Information and Communication Technology (ICT) and technology-based interventions (Cotterell et al., 2018) can support this endeavor (Goodman-Casanova et al., 2020) and progressively help people manage, cope with, or even treat a variety of physical and mental health issues (Berkowsky et al., 2015). ICTs have been found to have a more direct impact on older adults’ health and quality of life leading to decreased depression (World Health Organization (WHO), 2017) and loneliness (Cotten et al., 2013), participation in activities, boosting of self-confidence (Meiland et al., 2017) and empowerment of independent life (Hülür and Macdonald, 2020). On top of that, older adults today gradually become more involved in digital life (Zickuhr and Madden, 2012) while use of the internet and digital technology in general is also reported at increasing rates (Seifert and Schelling, 2015). Indeed, the rapid development of the digital era and the subsequent rapid growth of older adults who use technology has brought a new development face to the aging society (Ying and Zonghua, 2020) promoting in that way older adults’ digital re-socialization (Lapshin, 2018). Despite the challenges and difficulties older people may be confronted with, new technologies could be beneficial for older adults nowadays (Hülür and Macdonald, 2020). In this context, various ICT-based approaches and innovations have been introduced addressing the support of older adults’ care (Padilla-Góngora and Padilla-Clemente, 2008) and ensuring many advantages related to increased accessibility and flexibility, promotion of empowerment and increase of innovation (Lal and Adair, 2014). Among others, these services can be categorized as technological aids and ambient assisted-living systems providing support to older adults in certain daily life activities; cognitive assessment or cognitive interventions based on ICT providing cognitive and emotional support for older adults and their caregivers (Barnes et al., 2009; Franco-Martín et al., 2011; Donker et al., 2013; García Lizana, 2013; Boots et al., 2014; Lampit A. et al., 2014; Lampit A.H. et al., 2014; Xavier et al., 2014; Kauppi et al., 2015; Chan et al., 2016; Chen, 2016; World Health Organization (WHO), 2016; Herrero et al., 2020); and technologies and interventions aiming to enhance older people’s social participation through technology (Chen, 2016).

Various studies exploring the effectiveness of technology-based interventions in promoting the mental health and well-being of older people are reported in literature. According to WHO, technology-based interventions, i.e., interventions based on access and use of a technological device (e.g., computer, telephone) or process (e.g., internet, video), can potentially improve mental health and well-being of the older population, including those suffering from poor mental health (World Health Organization (WHO), 2016). Changes in depressive symptoms and satisfaction with the treatment were reported in a study implemented web-based interventions in a sample of 193 participants with depressed diagnosis (Herrero et al., 2020), while a more systematic review of eight studies exploring the use of mobile applications focusing on depression, anxiety disorders and substance abuse care found that apps have the potential to improve health outcomes and treatment accessibility (Donker et al., 2013). Furthermore, additional research has supported that access and interaction with electronic devices and telehealth services can help people suffering from mental health issues to manage and track their health indicators anywhere in the community (Kauppi et al., 2015), as well as maintain and enhance cognitive function (Chan et al., 2016).

The number of computerized cognitive training (CCT) programs has rapidly increased in the last few years. There is evidence showing that CCT improves overall cognitive function, memory, processing speed, working memory and visuospatial skills (Lampit A. et al., 2014). A recent randomized trial comparing CCT with an active control group found improvements in memory, global cognition, and processing speed that were maintained over a 1-year follow-up, suggesting retention of CCT benefits (Lampit A.H. et al., 2014). Therefore, CCT is a promising strategy to promote healthy cognitive aging and is also a viable strategy for those with limited ability to engage in other lifestyle strategies, including exercise.

In a large-scale longitudinal study on aging, it was found that over time, increased internet use was associated with significant improvement in delayed recall (Xavier et al., 2014). Other studies involve cognitive training programs/software based on new technological systems that provide older adults access to gamified, attractive, cognitive exercises everywhere and at any time and (Barnes et al., 2009; Smith et al., 2009). These exercises aim at improving overall functionality in areas including memory, attention, speed, executive functions, orientation, and cognitive skills (Frantzidis et al., 2014; Bamidis et al., 2015; Klados et al., 2016). Moreover, older adults, who use computers, have been observed to be at a lower risk of receiving a diagnosis of dementia up to 8.5 years later (Almeida et al., 2012).

Beyond doubt, the COVID-19 pandemic has set in motion waves of change with a wide range of possible trajectories (OECD, 2020) and has impelled many health and social care professionals to face with the new demands of the disease and, therefore, engage in a steep learning curve relating to the virus itself and its necessary operational changes (Fisk et al., 2020). In an effort to abide by these guidelines and provide the essential care to those in need, telemedicine, health care carried out at a distance (Wootton, 1995) and telehealth, technologies and related services concerned with health and well-being provided for people irrespective of location (Fisk et al., 2020), have emerged as crucial care delivery mechanisms aiming to protect caregivers and care recipients (Lam et al., 2020). As it has been designated by other areas of healthcare, implementing telehealth in the context of a pandemic is a valuable modality for providing essential care to the most vulnerable groups (Calton et al., 2020).

Our study underlines the impact of social isolation in general population, as well as the importance of empathic skills and active coping strategies in promoting the individuals’ psychosocial adaptation to a threatening event, like the COVID-19 pandemic. In this context, this piece of work aims to explore the effectiveness of potential benefits of a technology-based initiative during the first COVID-19 outbreak in Greece focused on older adults and people that belong in vulnerable groups. Indices of their quality of life and health status were studied before and after the intervention composed of a web-based cognitive training software.

## Materials and Methods

### Study Design and Participants

The non-randomized intentional (or purposive) sampling method was followed in this study. Intentional sampling method is a non-randomized procedure that is based on the recruitment of a particular group of individuals as participants of a study with the purpose of meeting specific prescribed criteria (Lauretto et al., 2012). Based on that the inclusion criteria were set as follows: people that belong in vulnerable groups (older adults ≥ 60 and people suffering from at least one health issue), fluency in Greek, internet access by phone, tablet or computer, time commitment to the training protocol, good hearing and sight as well as without having diagnosed with any significant mobility difficulties. On the contrary, exclusion criteria were: having diagnosed with severe neurological and communication disorders, drug abuse and concurrent participation in another study.

Participants’ recruitment was actualized within the network of the Integrated Health and Social Care System Long Lasting Memories Care – LLM Care ecosystem: municipalities and public entities, hospitals, rehabilitation centers and nursing homes as well as a great number of individuals/beneficiaries. Both direct and indirect recruitment strategies were implied where, in particular, members of the research team and LLM Care network’s healthcare professionals were responsible for the identification, approach and selection of participants who were eligible for participating in the study based on the inclusion criteria.

Initially, 148 potential participants against inclusion and exclusion criteria were identified and informed about the study. Among these, a total of twenty-four (24) participants met the inclusion criteria and were assigned as eligible to join the study. These participants were requited with a mean age of 69.3 ± 9.8 years and an average of 12.08 ± 3.84 years of education, while female (62.5%) and married (75%) being the majority of the participants (Table 1). In addition, all participants suffered from a number of health issues and related comorbidities. In particular, 66.7% suffered from neurodegenerative disorders (Parkinson’s disease, dementia, Alzheimer’s disease, encephalopathy, vascular stroke or chronic consequences due to previous stroke), 33.3% from musculoskeletal conditions (vertigo), 29.2% from cardiovascular diseases (hypertension), 16.7% from metabolic diseases (diabetes mellitus), 8.3% from respiratory diseases (asthma, chronic obstructive pulmonary disease (COPD), 8.3% psychiatric disorders (depression, bipolar disorder), and 37.5% from comorbidities. This intervention was conducted without the use of a control group.

**TABLE 1:** Demographics.

Demographics	Mean ± std
Age	69.3 ± 9.8 years
Gender	62.5% Females, 37.5% Males
Education (years)	12.08 ± 3.84 years
Marital status	75% Married, 4.2% Unmarried, 4.2% Divorced, and 16.7% Widowed
Number of sessions	32 ± 18

The protocol of the study was approved by the Bioethics Committee of the School of Medicine of the Aristotle University of Thessaloniki. Participants were initially fully informed about the scope and the purpose of the study, while specific instructions and guidance were also provided in respect to the cognitive training. In this context, a psychosocial support guide has been developed and distributed to participants for coping with psychosocial effects emerged from the adverse COVID-19 related circumstances including suggestions that may set the ground for building individuals’ “psychological resilience” (the term psychological resilience refers to individual’s positive adaptation of the person after a traumatic event, as well as in their ability to cope).

### Integrated Health and Social Care System Long Lasting Memories Care – LLM Care

The Integrated Health and Social Care System Long Lasting Memories Care – LLM Care (Llm, 2021), is an ICT platform that combines cognitive training exercises (Brain, 2021) with physical activity (wFitForAll, 2021) providing evidence-based interventions in order to improve both cognitive functions and overall physical condition (Bamidis et al., 2015) and, therefore, quality of life. The combination of cognitive and physical training provides an effective protection against cognitive decline as age-related, thus, improving overall quality of life through the enhancement of physical condition and mental health, while preventing any deterioration and social exclusion (Bamidis et al., 2015). LLM Care is considered as a non-pharmaceutical intervention against cognitive deterioration that provides vital training to people belonging to vulnerable groups in order to improve their mental abilities while simultaneously boosts their physical well-being through daily monitoring.

LLM Care has been recognized as an innovative ecosystem and was thereby awarded a Transnational “Reference Point 2 *” within the European Innovation Partnership for Active and Healthy Aging (EIP on AHA) (Bousquet et al., 2019; European, 2021) due to its excellence in developing, adopting, and scaling up innovative practices on active and healthy aging. LLM Care incorporates two interoperable components, namely the Physical Training Component and the Cognitive Training Component. The intervention of this study utilized only the latter component.

#### Cognitive Training Component

**FIGURE 1:**
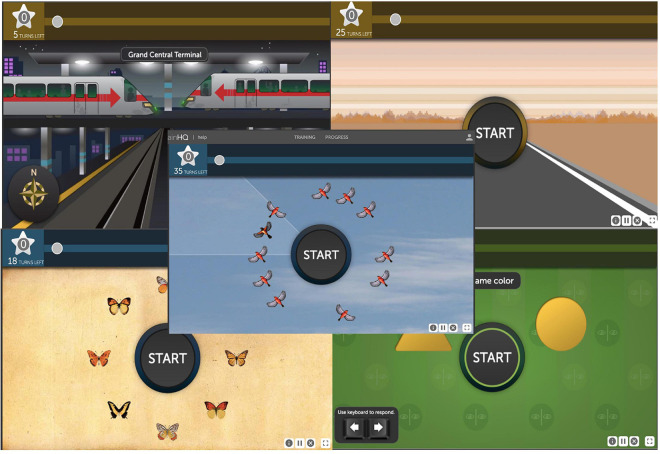
Screenshot from the Cognitive Training Component.

The cognitive training program (Figure 1) is based on the specialized software BrainHQ that was designed and developed by Posit Science in order to support cognitive game-based exercises in a fully personalized and adaptable cognitive training environment. Provision of personalized training, where each exercise is automatically adjusted to the participant’s level of competence, has been proven to accelerate and promote visual as well as auditory processing by improving memory, thinking, observation and concentration. It is an online interactive environment that incorporates highly empowering cognitive techniques and includes six categories with more than 29 exercises with hundred levels of difficulty, which focus on attention, memory, brain speed, people skills, navigation and intelligence. It is addressed to older adults, as well as individuals belonging to other vulnerable groups aiming at a healthier and more independent living.

### Intervention

**FIGURE 2:**
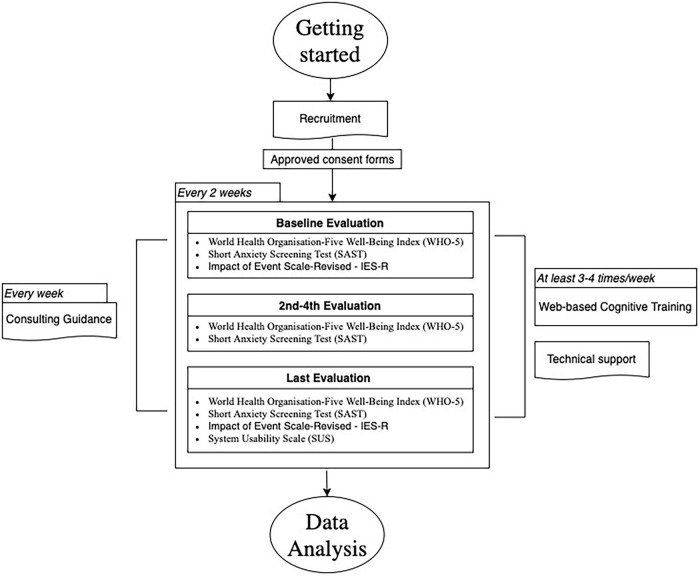
Visualization of the process followed with regard to the intervention.

The cognitive training software of the LLM Care was used in this study (with no cost), which is an interactive web-based training software to enhance participants’ cognitive functions by incorporating highly empowering cognitive exercises within a fully personalized and adaptable training environment. The ultimate goal of the interaction with the cognitive training was to promote the exercises as an original and new stimulus which, through the integration in the weekly routine of beneficiaries, will awaken and strengthen their cognitive functions, contributing to the improvement of their quality of life. The intervention lasted minimum 12 weeks from June to August 2020, with a frequency of 2–4 times per week in 30 min sessions (Figure 2), in which the participants interacted with the software by exploiting the exercises of their preference and using their personal devices (tablets/laptops) in their own residence. Additionally, participants were encouraged by trainers/researchers to fully exploit all the available exercises included in different categories aiming at improving their overall well-being and cognitive skills that are targeted to attention, verbal and spatial memory, visual processing speed, alertness, working memory, auditory perception and memory, processing speed, visual speed, precision, social cognition, facial recognition, intelligence, cognitive flexibility, as well as navigation skills. All cognitive exercises consisted of a great deal of levels and when initiating each exercise, a tutorial with essential guidance on the goal and type of interaction of each cognitive task was provided. The software itself included a special feature that allowed each exercise to adapt in difficulty as users worked so that they trained at the optimum level.

Consent forms along with psychometric assessments were digitally distributed to older adults every 2 weeks in order to periodically evaluate their psychosocial and mental health condition (five evaluations in total). Ten (10) LLM Care certified trainers, mostly consisted of psychologists, acted as remote facilitators during the intervention. Aiming to develop a more intimate and trusting relationship with each of the beneficiaries, each trainer was assigned to communicate every week with specific number of participants using digital means (telephone, e-mail, viber, skype) or other tools depending on participants’ needs and preference. In particular, weekly communication had a dual aim; (a) provision of technical support and (b) consulting guidance. Technical support included assistance in resolving technical problems, such as log-in or accessibility issues during the interaction with the cognitive training program. Consulting guidance was focused on the beneficiaries’ interaction with the cognitive training and their overall experience, aiming to gain the best possible cognitive benefits and maintain a friendly and supportive communication. In that way, participants had the opportunity to discuss and share their concerns and feelings with regard to the LLM Care intervention. In addition, monitoring of beneficiaries’ progress was available to trainers enabling the personalized and adequate provision of guidance and support to beneficiaries. Monitoring was based in features that allow visualization and mapping of data to the user record account, such as completed exercise levels, baseline and “best” scores in each exercise level, tracking of training through personal calendar and progress and performance chart.

It is worth noting, that the intervention was enhanced though supplementary material that was designed and developed aiming to alleviate the impact of the COVID-19 pandemic. In this context, two (2) COVID-19 Guides were also developed by the members of the research team aiming to provide both beneficiaries and associating entities with a series of guidelines and recommendations during the pandemic era. The “Beneficiaries’ COVID-19 Self-management Guide” was addressed to the provision of proper guidance for the effective implementation of the cognitive training and consultation to beneficiaries during the period of the pandemic, while the “Associating Entities’ COVID-19 Management Guide” to the adaptation of the way the LLM Care intervention is conducted in associating entities’ infrastructures in accordance with the measures and regulations that were introduced during the pandemic.

### Measures

#### Impact of Event Scale-Revised – IES-R

The Impact of Event Scale-Revised (Weiss, 2007) is based on the Impact of Event Scale (Horowitz et al., 1979), which is one of the most commonly used measures regarding traumatic experiences. The IES-R, in particular, is a self-report scale about post-traumatic stress disorder, as it is in accordance with the DSM criteria for PTSD. It consists of 22 items, rating from 0 (not at all) to 4 (extremely), with respect to how distressing each of them has been during the past 7 days for the respondent. These 22 items are categorized into three subscales and the total score ranges between 0 and 88. The first one is intrusion including 8 items (regarding intrusive, thoughts, feelings, images or nightmares related to the traumatic event), the second one is avoidance (of related situations, feelings, etc.) which also includes 8 items and the third one is hyperarousal with 6 items (e.g., irritability, concentrating difficulties, etc.). As far as the Greek-language version of the IES-R (IES-R-Gr) is concerned, the relevant research has revealed that it is an instrument with satisfactory psychometric properties, appropriate for both clinical and research purposes (Mystakidou et al., 2007).

#### World Health Organization-Five Well-Being Index (WHO-5)

The World Health Organization-Five Well-Being Index (WHO-5) is a short self-reported measure of current mental well-being. The assessment was initially introduced in 1998 by the WHO Regional Office in Europe (WHO, 1998) in the context of the DEPCARE project on well-being measures in primary health care. The WHO-5 is a self-report assessment that consists of five statements, using a 5-point Likert scale ranging from 0 “At no time” to 5 “All of the time,” in relation to the past 2 weeks, where the raw score 0 represents low levels of quality of life and 25 represents greater quality of life (Topp et al., 2015). Example items include “I have felt cheerful in good spirits,” “I have felt active and vigorous” and “I have felt calm and relaxed.” The scale has been widely used among Greek populations, showing high reliability scores and, therefore, validating the Greek language translation of the scale (Papanas et al., 2010).

#### Short Anxiety Screening Test (SAST)

The scale is a clinician short rating scale for detection of anxiety in older people, consisting of 10 items rated on a 4-point response scale (“rarely or never,” “sometimes,” “often,” “always”) generating scores between 10 and 40, with higher scores meaning higher levels rate on anxiety scale. A score that is under 24 is a cut-off point score for existence of an anxiety diagnosis and a 22–23 is a borderline score. In general, the SAST questionnaire showed a very good overall internal consistency (a value: 0.763, 95% CI 0.71 to 0.82, P < 0.001) for individual comparison. The overall Cohen indicator for reproducibility (test-retest reliability) is categorized as “very good” (0.930, 95% CI 0.918 to 0.942, P < 0.0001). The Greek version of the test is a suitable version for Greek primary healthcare system and is a valid tool for screening anxiety symptoms in older people, with a good internal consistency, and high test and retest reliability (Grammatikopoulos et al., 2010).

#### System Usability Scale (SUS)

The System Usability Scale (SUS) is the most frequently used standardized questionnaire to measure perceived usability. It was developed by John Brooke in 1986 (Brooke, 1996) and it helps evaluate a wide variety of products and services, including hardware, software, mobile devices and websites. It includes 10 items with five response options ranging from Strongly Agree (1) to Strongly disagree (5) (Lewis, 2018). Example items of the scale include “I think that I would like to use this system frequently,” “I thought the system was easy to use” and “I felt very confident using the system.” The SUS scale has been validated and standardized in the Greek language and has been exploited among Greek populations (Katsanos et al., 2012).

## Data Analysis

After the completion of the data collection stage, the responses were downloaded from Google Forms, updated in Microsoft Excel and transformed to SPSS Statistics 24 for further statistical tests. Descriptive statistics, including means, counts, and percentages for the variables, were calculated. To assess the reliability of SUS, WHO-5, SAST and IES-R scores, the internal consistency of each score was measured by means of Cronbach’s alpha. A value between 0.7 and 0.9 was regarded as satisfactory.

Correlation analysis was conducted between the IES-R total score (including the 3 subscales), SAST score and WHO-5 score. More specifically, correlation coefficients were used in order to examine associations between each score and the variables of gender, age, education and marital status separately. Results refer to participants who completed all evaluations properly. For all analyses, a two-tailed *P*-value < 0.05 was considered statistically significant.

## Results

### Descriptives

**FIGURE 3:**
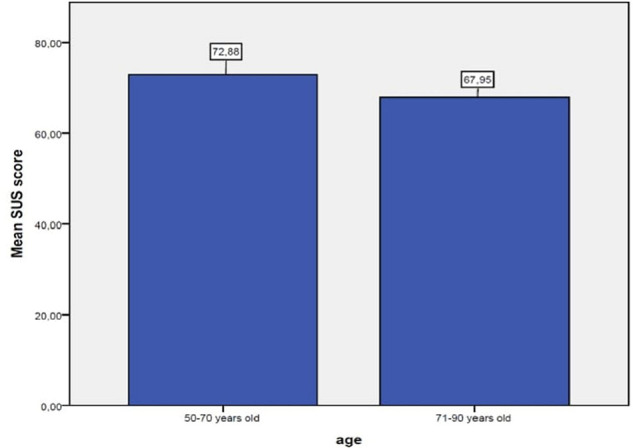
SUS score mean by age.

The average SUS satisfaction score was closer to 70 (70.6 ± 17.07), indicating an acceptable experience. Participants, between 50 and 70 years old rated higher the usability (72.88) compared to the participants between 71 and 90 years old (67.95) (Figure 3). For WHO-5, the mean value (± standard deviation) reported 59.50 ± 26.04 for baseline, 71.50 ± 19.96 for the 2nd evaluation, 68.50 ± 20.64 for the 3rd evaluation, 66.73 ± 21.50 for the 4th evaluation and 68.00 ± 23.71 for the last evaluation (Table 2). The mean values (±standard deviation) for total IES-R-Gr score and the 3 subscales are reported at Table 3.

**TABLE 2:** Cronbach’s alpha, mean, standard deviation of WHO-5 evaluations.

WHO-5	Cronbach’s Alpha	Mean ± SD
Baseline evaluation	0.882	59.50 ± 26.04
2nd evaluation	0.843	71.50 ± 19.96
3rd evaluation	0.849	68.50 ± 20.64
4th evaluation	0.847	66.73 ± 21.50
Last evaluation	0.899	68.00 ± 23.71

**TABLE 3:** Alpha coefficients, subscale means, and test-retest reliability of IES-R subscales.

Subscales	Cronbach’s Alpha	Mean ± SD	Pearson’s correlation coefficient
Intrusion	0.74	0.67 ± 0.34	0.404^a^
Avoidance	0.78	0.91 ± 0.35	0.519^a^
Hyperarousal	0.63	0.79 ± 0.70	0.377^a^

*SD = standard deviation.*

*^a^*P* < 0.05.*

### Reliability

#### Internal Validity

The Cronbach’s alpha for SUS was 0.752 (10 items), which indicates that the answer for each question is consistent with the others, yet they do not overlap. The Cronbach’s alpha for WHO-5 was 0.882 for the Baseline evaluation, 0.843 for the 2nd, 0.849 for the 3rd, 0.847 for the 4th and 0.899 for the Last evaluation, respectively, which indicates that the answer for each question for the five evaluations is consistent with the others (Table 2). For IES-R score, to estimate the internal consistency, coefficient alphas were calculated for the avoidance, intrusion, and hyperarousal subscales. The results produced the following coefficients: intrusion, Cronbach’s *a* = 0.74; avoidance, Cronbach’s *a* = 0.78; and hyperarousal, Cronbach’s *a* = 0.63, while overall alpha = 0.88 (Table 3).

#### Test-Retest (Stability)

Data from the 24 participants who responded both at the first and the last assessment of the study was used to examine the stability of the IES-R over time. The test-retest correlation coefficients (Pearson’s rho and intraclass correlation coefficients) indicated that all IES-R-Gr scores were remarkably consistent across the two occasions and were significantly correlated (Table 3).

### Validity

#### Construct Validity

As normality was met, Pearson correlation indicated a negative correlation between age and SUS score (*r* = −0.483, *n* = 24, *P* = 0.017). Furthermore, Pearson correlation was used to examine associations among the initial and last evaluations of IES-R total score including subscales, SAST and WHO-5 scores. The results showed a significant correlation between WHO-5 and IES-R total scores (*r* = −0.544, *P* = 0.006), WHO-5 and hyperarousal scores (*r* = −0.622, *P* = 0.001) and WHO-5 score and intrusion scores (*r* = −0.432, *P* = 0.035) (Table 4). A significant association was also found between age and WHO-5 (*r* = 0.424, *P* = 0.039) (Table 4). Lastly, no significant correlations were found between SAST scores and age, as well as with the other psychological assessments.

**TABLE 4:** Correlation coefficients between WHO-5 and IES-R scores, including subscales.

	WHO-5 score	*P*-value	Age	*P*-value
*IES-R total score*	*r* = −0.544	**0.006**	*r* = −0.170	0.427
*IES-R intrusion*	*r* = −0.432	**0.035**	*r* = −0.371	0.074
*IES-R avoidance*	*r* = −0.358	0.086	*r* = 0.056	0.794
*IES-R hyperarousal*	*r* = −0.622	**0.001**	*r* = −0.160	0.456
WHO-5	–	–	*r* = 0.424	**0.039**

*The bold values indicate the statistical significance with *p* < 0.05.*

## Discussion

The aim of the current study was to explore the impact of cognitive training intervention on the well-being of older adults and people that belong in vulnerable groups through the exploitation of an interactive web-based software. In this context, the cognitive training software of the Integrated Healthcare System LLM Care was used in this study as an online tool that provides evidence-based, personalized intervention for improving the level of cognitive functioning in older adults and promoting their overall well-being.

Findings of this study indicate that older adults with improved well-being tended to report decreased subjective distress caused by the traumatic COVID-19 related consequences. In particular, facing the unprecedented and adverse circumstances emerging from the COVID-19 pandemic, may be appraised as a psychological distressing event causing negative side effects especially in more vulnerable population, such as older adults, due to their unique psychosocial and physical needs (Bailey et al., 2021; Giordano et al., 2021). The lack of face-to-face interactions along with the additional containment measures that globally affected participants’ overall well-being and mental health worsen the cognition and functioning of this population (Goodman-Casanova et al., 2020). More specifically, they limited their social interactions even with family members, and this highly increased their loneliness and anxiety levels due to quarantine/self-isolation and the uncertainty of the outbreak. Thus, the restrictive measures contributed to the rise of psychological symptoms such as depression, anxiety, anger, and subjective cognitive failures (Maggi et al., 2021). To this end, it has been widely highlighted that home-based assistive technology interventions (Cotterell et al., 2018) can support and improve the quality of life and wellbeing of vulnerable people during the COVID-19 pandemic, while enhancing their cognitive abilities and mitigating the negative outcomes caused by the current stressful situation. Following this perspective, the negative outcomes of intrusion such as intrusive thoughts and feelings regarding the distressing situation and hyperarousal symptoms, for instance anger and irritability are minimized, while their overall well-being is increased during their interaction with LLM Care. Vulnerable groups of people, such as older adults, could benefit in several domains of their wellbeing from the LLM Care platform, without the barrier of negative intrusive emotions and thoughts that would compete with the impact of the intervention benefits. In conclusion, in times of crises, such as a pandemic, older adults and vulnerable people may benefit from the exploitation of the LLM Care platform, through reducing to some extent their negative thoughts and feelings that result from the adversities generated by the pandemic circumstances. In addition, older adults’ cognitive status declines as years pass by, while an interesting observation was made that their emotional regulation was increased. Older adults seem to improve their emotional regulation skills (Birditt and Fingerman, 2005). Based on the literature, getting older involves a decrease in the perception of negative affect and there is a correlation with a stabilization or even a slight increase in the perception of positive affect (Carstensen et al., 1999). One theory that tried to explain better emotional regulation during the process of aging was socio-emotional selectivity theory (Smith and Hollinger-Smith, 2015). According to that theory, the lifetime perspective changes the motivations of older adults and well-being is more important. Other theories are focusing on the emotional processing and the tendency of older adults to show a preference for positive information. Moreover, high resilience in older adults has been associated with reduced depression and mortality risk, as well as with better perception and successful aging, leading to an increased quality of life (De Raedt and Ponjaert-Kristoffersen, 2006; Jeste et al., 2013). These findings are in line with our hypothesis that well-being seems to remain relatively stable among older adults and decreases only when adversities occur and agree with the theories of self-immunizing processes. An experimental study that studied older adults in a real-life stressful situation also showed that decreased physical resources are related to a realistic appraisal of performance and less depressive feelings (Mroczek and Kolarz, 1998). Overall, it is important to consider that the systematic contact among participants and the research team for technical and consultation purposes may have offered a significant impact on the deployment of the cognitive training program by vulnerable people and consequently, potentially influenced their overall well-being levels. Following this perspective, this systematic contact could be perceived as an external factor that might have resulted in participants feeling more secure, engaged, motivated and encouraged when exploiting the program, as trainers were young people with high levels of education and deep knowledge of cognitive training protocols among vulnerable people.

In addition, participants of the current study, due to suffering from comorbidities of diseases, are potentially included in vulnerable groups. Specifically, vulnerability is associated with a sense of low quality of life and a possible indicator for this is frailty. Frailty is not only related to the biological process of aging but also to the progress of the disease and as an indicator is associated with the development and progress of the disease. Therefore, frailty is not exclusively associated with individuals’ chronological age but with their fragility and deterioration of conditions even in younger age groups (Ribeiro et al., 2020). This could be further explained as a risk factor, which relates to individuals’ reduced ability to deal with stressors resulting in the increase of the vulnerability of these groups (Mitnitski et al., 2001; Quinlan et al., 2011; Kojima et al., 2019). Frailty has also been linked to cognitive impairments, depression (Robine et al., 2019) and poor social networks (Sakurai et al., 2019). The role of social networks is highly important as it plays a protective role in individuals’ cognitive functionality (Holtzman et al., 2004; Crooks et al., 2008; Bae et al., 2018) and, therefore, poor social network is associated to social frailty and (Sakurai et al., 2019) increased vulnerability (Bae et al., 2018). Therefore, it is essential to provide effective interventions to reserve frailty (Cunha et al., 2019). To this end, the cognitive training intervention presented in the current study aimed to enhance the well-being and quality of life of people that belong in vulnerable groups during the COVID-19 situation, when their social networks were limited, through minimizing risk factors that could potentially hasten the progress of their disease.

Regarding the usability of the software, the outcomes revealed that it was marginally acceptable by the participants. In fact, it was observed that the usability ratings of the platform tended to be decreased from the participants who were older, something that probably indicates the difficulties that older adults experience when engaging with new technologies. This is a finding highlighted, also, from other studies, which showed that possible barriers concerning the use of technology from older individuals are related with negative attitudes, lack of knowledge and experience (Gitlow, 2014; Meiland et al., 2017), as well age-related changes such as vision impairment, hearing loss and greater limitations in memory and physical capacity (e.g., fine motor difficulties, disability) (Papanas et al., 2010; Topp et al., 2015). In addition, a number of studies have reported a correlation between technology use from older adults and a general negative assessment of their own skills (Gitlow, 2014; Gell et al., 2015), as well as greater fear and anxiety when using computers and technology in general (Marquié et al., 2002). Moreover, another important factor that has been designated in the existing literature as relevant with older adults’ engagement with new technologies is the social environment that those people have and the availability of support, which also seems to affect how they interact with technology and how they experience possible difficulties (Barnard et al., 2013).

Possibly, the exploitation of LLM Care increases older adults and vulnerable people’s capabilities to a number of aspects of their lives, providing them with a sense of control over their external and internal environment and helping them to better compensate with adversities that challenge their lives. The compensation also acts indirectly by strengthening the already existing individuals’ capabilities that arise as a result of age-related changes, such as the shift to mental reserves or other existing individual variables that have a positive effect on the quality of life, such as marital status or other personality factors. During the pandemic, the LLM Care interventions may have acted as a substitute for daily living activities and aspects of life that traditionally people use to increase their well-being.

## Limitations and Future Directions

Important limitations of the current research are mainly related to the COVID-19 restrictions, resulting in the requitement of a small sample of participants and in the lack of a controlled common setting. The small sample size was a significant factor that did not allow the conduction of analysis focusing on the interaction of COVID-19 and pre-post factors, considering the small and uneven sample subgroups that arose.

Another limitation is related to the duration and period of exposure to the intervention. More specifically, it could be argued that 12 weeks is considered too short to cause substantial changes to participants’ overall well-being. The research was conducted during summertime, which was the interval of the two pandemic waves in Greece and, therefore, participants’ mood and daily life was potentially directly or indirectly influenced by other external variables, such as the social and environmental circumstances.

An additional limitation of this study is the absence of a control group. In this study we present the results of preliminary statistical analyses performed on exploring the impact of cognitive training intervention on older adults’ well-being from ongoing research. While this paper is part of an ongoing study, further data collection and involvement of a control group is needed to assure further accuracy on the results and is hoped to be obtained through the continuation of this study.

Moreover, further limitations of the current study correspond to the fact that participants’ overall wellbeing levels might have been increased over the course of the cognitive training intervention due to additional external factors. Following this perspective, it is important to take into consideration any external factors that can potentially influence participants’ wellbeing during the intervention. In this case, such indirect external factors can be considered the intimate social contact among participants and the research team, while exploiting the cognitive training software. To this end, the identification and exploration of such external factors influencing participants’ wellbeing is considered essential in a future larger scale study, along with the addition of a control group that will not take part in the cognitive training intervention and will only interact with the research team.

Future directions could focus on extending the duration of the intervention, in order to designate long-term effects and outcomes. In addition, control groups of participants in the same or different age range could also be included in the study to make comparisons among different groups of people through the exploitation of the LLM Care platform. Finally, a mixed design methodology, which will include both quantitative and qualitative research methods, such as observation or semi-structured interviews that will require participants to define and elaborate on the impact of the engagement with the LLM Care platform in their daily lives.

## Conclusion

The COVID-19 crisis has been identified as a major public health concern that generated adverse outcomes in multiple domains of individuals’ daily living. Health-related factors, such as the presence of several diseases, place individuals at a greater risk for vulnerability, which can be a significant risk factor for frailty across the life continuum, regardless of chronological age. In this context, the state of vulnerability is a condition that can often accelerate physical and cognitive decline, undermining, that way, the process of active aging as well as the overall well-being of individuals. In particular, this crisis has created significant barriers to maintain safe conditions for social interactions among individuals, especially for older adults, who belong in vulnerable populations. This issue has resulted in causing distressing emotions among older adults and, thus, enhancing loneliness, anxiety and reducing their well-being.

There is evidence that interventions incorporating new assistive technologies, such as the LLM Care platform, enable vulnerable populations, namely older adults to improve aspects of their quality of life and enhance their well-being through web-based cognitive training. In particular, the improvement of their cognitive abilities might reduce the impact of psychoactive conditions and factors, for instance the COVID-19 pandemic, and make a significant contribution to support these vulnerable populations, who have been affected on a psychosocial, physical and mental level. In conclusion, the exploitation and engagement with the LLM Care platform contributes to the improvement of vulnerable people’ well-being and alleviates the negative experience caused by distressing conditions like COVID-19.

## Data Availability Statement

The data that support the findings of this study are available from the corresponding author, upon reasonable request.

## Ethics Statement

The study protocol was approved by the Bioethics Committee of the Medical School of the Aristotle University of Thessaloniki and was conducted per the Helsinki Declaration of Human Rights. The participants provided their written informed consent to participate in this study.

## Author Contributions

ER, VZ, and PDB contributed to conception and design of the study. SG, ID, VP, and A-MK implemented the intervention and collected the data. SG performed the statistical analysis. ER, VZ, SG, ID, AV, VP, A-MK, and MK wrote sections of the manuscript. PDB supervised the manuscript. All authors contributed to manuscript revision, read, and approved the submitted version.

## Conflict of Interest

PDB discloses potential (non-financial and beyond the context of the submitted work) conflicts of interest with Posit Science: there is a co-marketing agreement between the company and the Aristotle University of Thessaloniki to exploit BrainHQ within the LLM Care self-funded initiative that emerged as the non-for-profit business exploitation of the Long-Lasting Memories (LLM Project) originally funded by the ICT-CIP-PSP Program of the European Commission. BrainHQ now forms part of LLM Care, a technology transfer/self-funded initiative that emerged as the non-for-profit business exploitation of LLM. The remaining authors declare that the research was conducted in the absence of any commercial or financial relationships that could be construed as a potential conflict of interest.

## Publisher’s Note

All claims expressed in this article are solely those of the authors and do not necessarily represent those of their affiliated organizations, or those of the publisher, the editors and the reviewers. Any product that may be evaluated in this article, or claim that may be made by its manufacturer, is not guaranteed or endorsed by the publisher.
